# How to earn a reputation as a great partner

**DOI:** 10.5195/jmla.2018.504

**Published:** 2018-10-01

**Authors:** Betsy L. Humphreys

**Affiliations:** Arlington, VA

## Abstract

Boundary spanning is a core activity for health sciences librarians. To be effective, librarians must bridge internal silos and reach across borders to partner with other disciplines, groups, and organizations. Common sense strategies and practical implementation steps can help librarians to earn a reputation as a trustworthy and effective partner.

## INTRODUCTION

It is often said that change is the only constant. For librarians, however, there are a few other constants, including the perpetual need to partner with those outside our discipline and beyond our offices and home institutions to accomplish worthwhile goals.

As defined in a 2002 paper by Williams, “boundary spanning” is reaching across borders, margins, or sections to “build relationships, interconnections and interdependencies” in order to manage complex problems. Boundary spanning individuals build “sustainable relationships, manage through influence and negotiation, and seek to understand motives, roles and responsibilities.” Boundary-spanning organizations create “strategic alliances, joint working arrangements, networks, partnerships and many other forms of collaboration across organizational boundaries” [1].

Boundary spanning is obviously a core activity for health sciences librarians. To be effective, libraries must bridge internal silos and reach across borders within the larger institutions that they serve, as well as with outside groups, disciplines, and organizations.

In my view, successful boundary spanning depends on a “systems approach” to solving problems and embracing new opportunities. In other words, use a broad perspective to identify all elements that contribute to something that is less than ideal or, alternatively, that is likely to be essential to building something new and wonderful. Think about how the parties currently or potentially involved with each key element might work together to make the world a better place and then engage with those parties to develop a feasible way forward. As many health sciences librarians have illustrated, the scope of the problems and opportunities to be considered for potential action should be at least as broad as the purposes and goals of the organization that the library serves.

In a long career at the National Library of Medicine, I found working across disciplines and organizations to be fascinating and transformative. It expanded my view of what should—and could—be done to improve access to biomedical and health information. Reflecting back on my partnership opportunities and experiences led me to identify some “things that worked for me” that may be useful to others who are reaching across boundaries to accomplish important goals.

## PRACTICAL STEPS

I offer these thoughts on the assumption that most health sciences librarians already have the foundation needed to be great partners: they work hard, persevere to achieve important goals, are interested in the wider world in which they operate, and are both competent and trustworthy. What follows are common sense approaches and practical implementation tips designed to build on that foundation. They will be familiar to many. Of course, nothing guarantees success or is appropriate in all cases, but these approaches often produce positive results.

### Follow the “golden rule,” but aim for “platinum”

It should go without saying that, at a minimum, you should treat current and potential partners as you wish to be treated. This includes assuming that they are intelligent, well-meaning people with useful expertise and experience who are attempting to accomplish good things within the parameters of their organization’s mission and available resources. If their behavior occasionally does not make sense or seems counterproductive, your starting assumption should be that there has been a misunderstanding, a miscommunication, or a mistake—not that your partners are irrational or unreliable. Just as you hope that your partners will be a source of useful intelligence, will critique your ideas honestly, will devote serious thought and some energy to your joint endeavors, will support in public the positions they have espoused in private, and will follow through on commitments large and small, you should do the same for them.

If you are lucky, initial discussions and joint endeavors can lead to long relationships that will give you the insights necessary to treat partners as they wish to be treated. Greater familiarity allows you to use their preferred styles of communication and to provide the types of intelligence that your networks supply more reliably than theirs. It is especially helpful to learn the things that do not bother you but do bother your partners and to know some of their interests that are separate from your joint agenda. The latter can be a great learning opportunity for you and provide the seeds for future collaborations.

### Be prepared: have informed questions and ideas for joint action ready for the first encounter

Many meetings with people from other parts of your organization, from other disciplines, or from outside organizations are potential opportunities to broaden your perspective, to identify new joint endeavors, or to advance your current priorities. They also provide a chance to raise your profile as a credible and effective partner.

Experience has taught me that even a little preparation before a first meeting with new people, or on a new topic, can lead to support for or expansion of a current priority or goal and help you to gain new allies. A good first impression can be the start of something very big.

Gathering background intelligence and thinking about whether and how a new group or new topic might help to achieve some desirable goal prepares you to ask insightful questions and make substantive suggestions for potential action in that first meeting. Often, other meeting attendees will hang back to “wait and see” what happens. That can be a safer tactic in some circumstances, but taking a more proactive approach can allow you to learn more and gain influence over next steps. In any case, you may stand out as someone who thinks and makes constructive suggestions. Of course, if you sense that your preliminary ideas do not fit the situation after all or could lead to unwelcome assignments or outcomes, keep quiet.

Here are a few questions that are often worth a little research and thought before an encounter with new potential partners or on a new topic:

#### Is anyone else in your shop or your larger organization already working with this group or on this topic?

Even in a small organization, it can be dangerous to assume that you would know if they were. It is never impressive to potential partners when the right hand does not know what the left hand is doing. A quick email is often sufficient, and your colleagues will usually be pleased to be asked about any background knowledge, interactions, and ideas for productive collaboration that they may have. Their input may help you avoid landmines or, even better, build on previous positive collaboration.

#### Are there relevant numbers to review, such as data on budgets, revenue, employees, patients, students, publications, grants, clinical trials, or parking spaces?

What is the trajectory of those numbers: up or down? Are they likely to change due to developments on the horizon? Knowing relevant numbers and thinking about what affects them can help ground your questions and ideas in reality. If you cannot find relevant numbers, that may be the first insightful question to ask. If nobody has the data, getting them may be the best first joint task (e.g., Read et al. [2]).

#### Are there documents that should be reviewed prior to the meeting, such as the mission of the other organization, funding announcement, law, policy, regulation, or memo that underpins the topic of the meeting?

Reading the fine print may allow you to steer the conversation away from irrelevant topics. Do this tactfully, maybe even before the meeting. A short email with an attachment—“if you haven’t seen this, I found it useful as background for our upcoming discussion”—can save everyone time and gain you admirers. If you think there must be relevant documents but cannot find them, this would be another insightful question to ask.

### Know and be able to explain the “systems sense” of your activities and ideas

Working on something that makes no “systems sense” to current or potential partners damages credibility and is hard on those doing the work. Unfortunately, what does or does not make systems sense is very context dependent. The fact that your stakeholders do not understand why you are doing something is an obvious warning sign. Lack of use, duplication of effort, and failure to adopt or adapt to new systems, standards, resources, and tools are also danger signals. On the other hand, a rational and likely sustainable division of labor among interested and affected parties is often a hallmark of systems sense. Invading occupied territory or developing something to meet a need that is already well met by another entity is questionable but can be appropriate if the landscape is about to undergo radical change. Exploring the dimensions of a problem or devising tests or experiments to determine potential feasibility and utility are usually “systems sensible.”

In our world, systems sense is a rapidly moving target. Changes in technology, external information services, user needs and expectations, laws, and regulations will inevitably make some activities that were once very sensible—perhaps even brilliantly innovative and useful—seem unreasonable now. The history of health sciences librarianship is full of groundbreaking local and regional services that were later overtaken by more sustainable national systems. When the web browser arrived, virtually all previous interfaces to information systems made no sense, but it took time to change them all. Everything cannot be fixed at once. Some things have to wait, while higher priorities are addressed.

There is no shame in having a residual activity that no longer makes obvious systems sense, but there is danger if your partners and stakeholders notice it before you do. When a question arises about why “that” continues when it is no longer necessary or there is a more effective approach, your credibility will be enhanced if you have already reviewed it and have a cogent answer, such as a timetable for changing it or a compelling reason why change is not yet desirable.

If stakeholders raise the systems sense question about something you have not yet reexamined, all is not lost. You can retain your credibility by thanking them for bringing it up and then getting back after you investigate (preferably quickly), regardless of whether it is sensible to change anything.

In the opposite case, you and your partners may be attempting something innovative and potentially highly valuable, but your stakeholders and potential partners do not understand why or how it will lead to something useful (e.g., Humphreys et al. [3]). Possible ways to address this situation are changing your messaging based on feedback from confused people, working hard to get at least something working quickly, and providing incentives to enlist some users who then may become advocates. It is also important to be on the lookout for external developments that can strengthen the rationale for what you are already trying to accomplish, as well as noticing environmental changes that may reduce or eliminate its systems sense.

### Approach a potential partner with something concrete and probably doable

Having systems sensible ideas ready when unsolicited opportunities to forge new partnerships arise is great, but, of course, you often have to create opportunities to engage with potential partners. In such cases, arranging a meeting about “cooperation” in general is usually less effective than a contact about a specific, and to start with, relatively circumscribed matter. Addressing larger issues is easier once some credibility has been established.

It can make eminent systems sense for the library to be a key partner in achieving knowledge and data management objectives for its parent organization. When the organization’s aggregate volume of data sharing, data management, knowledge synthesis, and impact analysis tasks is great, but no one individual is required to perform these tasks frequently, some centralized support and compliance monitoring is likely to be an effective and efficient approach. It is wonderful if the organization immediately thinks of the library as an obvious locus for matters related to knowledge and data management. However, if they do not, then obviously, the library has to approach them.

Results may vary, but some common approaches often work as opening steps in establishing or expanding useful partnerships:

#### Take advantage of “newness.”

A “newness” factor—the implementation of a new system, a new regulation or requirement (e.g., Rosenzweig et al. [4]), a new funding opportunity (e.g., Williams and Rambo [5]), or a recently arrived, promoted, or assigned person—provides a ready-made rationale for reaching out to potential partners. If you devote a little thought to it, you can usually identify a substantive newness factor as a plausible reason for contacting any potential partner, whether internal or external.

#### Solve an obvious problem

A newness factor often provides the fresh perspective that identifies any lack of systems sense in current operations; in other words, existing problems that can and should be fixed. New staff members or people with new assignments in the library can be the newness factor that highlights fixable problems, if they are encouraged to question things that do not make sense and if their ideas receive serious attention. Something that is clearly problematic for your unit and for an external party provides a great opportunity for initiating contact.

#### Explore an adjacent possible

Another effective approach to bridging divides involves the expansion of the activities of an already strong partnership, as in Kauffman’s notion of expanding into the “adjacent possible” [6]. He was thinking of biospheres, but others have seen this as a metaphor for how to escape perceived limits and promote innovation. (e.g., Johnson [7]). It is an apt description for what health sciences librarians are doing to embrace new roles and challenges in the research data arena. As Kauffman expressed it, once you expand into the “adjacent possible,” you increase “the diversity of what can happen next” [6]. This is an elegant way of saying that one thing leads to another or that you should find a propitious environment to try something new. It can be easier to cross into new territory with current partners, and it is sensible to start where there is a better chance of success.

### Communicate clearly: provide actionable input, reliable support, and apologies, if appropriate

Here are a few reminders about how to translate the perpetual need for better communication into concrete, positive action:

#### State the obvious, diplomatically

Being clear about basic assumptions and providing essential background is an important strategy in any attempt at clear communication. It is especially critical in efforts to span boundaries or bridge divides. What is obvious on one side of the divide might not be obvious—or even true—on the other side. It is important to identify and clear up misunderstandings as soon as possible. However, when stating anything in any context, it is best NOT to imply that you are telling people something they do not already know. No matter what the topic, someone receiving the information could be an expert on it. If it is well known to a whole group, you will lose credibility. There is no downside to prefacing remarks with “to state the obvious” or “as you probably know” or sending information in a message that begins with “Just in case you have not already seen this.”

It can also be important to RE-state the obvious (again diplomatically), for example, by repeating in your committee’s report important recommendations from previous reports that have yet to be implemented. There is a natural inclination to say something “new” rather than repeat critical points that have already been emphasized by other groups, but this is dangerous. It does not take much space to remind people of the importance of previously stated, but as yet un-implemented, recommendations. Repetition may influence action, and the lack of it can lead to the erroneous assumption that you disagree with previously expressed priorities.

When dealing with current or potential partners, make sure that they understand the full ramifications of what is being proposed or planned, even if glossing over some points might reduce their opposition to something you favor. Few things demonstrate your trustworthiness better than a full explanation of the broader implications and potential downsides of a course of action that you wish to pursue. You will certainly lose credibility if partners later suspect that you suppressed potential ramifications to gain their agreement.

#### Provide information and input where, when, and in a form that is most likely to have an impact

Provide ideas, comments, or complaints to those who may be able to do something about them. If there is a potential landmine, try to warn partners about it before a meeting in which explosions may occur. If there is a deadline for input, get yours in on time and provide it in a useful format. “This needs more justification” is not as useful as “I suggest that you add that it will enable compliance with the X regulation and is likely to reduce the cost of Y.” If you are suggesting changes to something that must be finished very soon, make specific suggestions for very brief wording changes. If you do, your input is much more likely to be reflected in the final document.

#### Notify, explain, and express regret when directions change or commitments cannot be met

A person who never changes a previously announced direction has never had a new boss, has ignored important changes in the environment, or has not reassessed the systems sense of current activities. Changes in direction occur. When they do, obviously you should clearly communicate the change to partners and, to the extent reasonable, the rationale for it. You should also express regret for any inconvenience or extra work it may cause them. Remember that it is always possible to do this without pointing fingers at your superior or colleagues. That is almost never a good strategy.

In the same vein, sometimes you cannot get input in on time or meet other types of commitments to partners. Saying nothing when this happens can damage your reputation. It is naïve to hope that no one will notice. Obviously, if possible, you should alert people in advance of the deadline and ask if a late submission would still be useful. If that is not possible, apologize afterwards. If apologies start to mount up, it is probably time to rethink the number and type of commitments you are making.

### Practice “aggressive patience” when it really matters

Henry David Thoreau was correct that “he who travels with another must wait until that other is ready” [8]. However, in health sciences librarianship, virtually everything worthwhile involves traveling with others. You must be patient, but you can practice “aggressive patience” to hasten partners’ readiness when the destination is really important. Being aggressively patient involves such things as taking minutes, developing agendas, scheduling meetings, drafting documents, turning everything around quickly (no matter how long it sat on someone else’s desk), actively looking for new allies and opportunities to advance the cause, revising the means but not the goal (as long as the goal survives those periodic reassessments of systems sense), allocating resources, advocating for resources on behalf of your partners, and not giving up, no matter how long it takes. This is hard work, so the goal should be very systems sensible and worthy of a great deal of effort. Tenacity in pursuing a goal that has logic on its side can be a powerful force.

## CONCLUSION

Few things are as exhilarating or rewarding as working with, learning from, and addressing important objectives with people from other disciplines, other professions, other departments, other institutions, or other countries. Based on my experience, following some or all of these steps is likely to improve the process, the outcomes, and your reputation as a great partner.
